# Stage 1 Registered Report: The experiences and perceptions of parent-child interaction therapy for parents of young children with communication difficulties: A qualitative evidence synthesis protocol

**DOI:** 10.12688/hrbopenres.12974.2

**Published:** 2020-05-20

**Authors:** Ciara O'Toole, Rena Lyons, Donna Ó’Doibhlín, Fia O’Farrell, Catherine Houghton

**Affiliations:** 1 Department of Speech and Hearing Sciences, University College Cork, Cork, Ireland; 2 Discipline of Speech and Language Therapy, National University of Ireland, Galway, Galway, Ireland; 3 Boston Scientific Library, University College Cork, Cork, Ireland; 4 Down syndrome Ireland, Cork, Ireland; 5 School of Nursing and Midwifery, National University of Ireland, Galway, Galway, Ireland

**Keywords:** Parent-child interaction therapy, Experiences and perceptions, Speech, language and communication difficulties, Systematic review, Qualitative Evidence Synthesis

## Abstract

**Background:** Parent-child interaction therapy refers to a group of interventions mediated by trained parents to address areas of developmental difficulties in children. In the field of speech and language therapy it is used in early intervention for children with speech, language and communication difficulties. The intervention involves training parents and caregivers on the importance of responsivity and language input in daily interactions and coaches them on strategies to implement these with the children. As the success of the intervention is heavily influenced by caregiver engagement, understanding and acceptance, it is important to consider their views. However, to date there has been limited work on synthesising parental views of this intervention.

**Methods: **This is a protocol for a qualitative evidence synthesis of peer-reviewed qualitative papers addressing the experiences and perceptions of parent-child interaction therapy for parents of children with communication difficulties. We will complete a systematic search of 11 databases, review the reference lists and complete a cited reference search of all included studies. Two authors will independently screen tests for inclusion, initially by title and abstract, with full-text screening as necessary. Thematic synthesis will be used for all included studies. We will appraise the quality of included studies using CASP and confidence in the review findings using GRADE CERQual.

**Discussion:** As the views of parents are pivotal in the success of this intervention, the findings from this synthesis should  help to guide best practice and policy for the future implementation of parent child interaction therapy for children with communication difficulties..

## Background

### Speech, language and communication difficulties in young children

Children are said to have a difficulty with speech, language and/or communication when they are unable to listen, understand or speak in a developmentally appropriate way. Children born with conditions that are known to cause speech, language and communication difficulties, such as Down syndrome, are often identified early in development and so intervention begins early. Other children may not start to receive intervention until they are identified as having a delay or disorder in their language skills, often in the presence of age-appropriate milestones in other areas of development, such as motor skills. The benchmark of fewer than 50 words in their expressive vocabulary and failure to put two words together by two-years is often used to define late talking children ( Dollaghan, 2013; Fenson *et al.*, 1994). However, parents and caregivers may notice subtle differences earlier than this. For example, a child who fails to make or respond to eye contact or attend jointly with a caregiver to an event, toy or activity in the first year of life could cause parents to become concerned. Likewise, a child who does not smile, point or use gesture to act out what they want or express a feeling early into the 2 ^nd^ year, may be identified early from screening of communication difficulties by a health visitor. There are also known risks associated with social-demographic factors (e.g. gender or socioeconomic status), family history, parenting and child behaviour that are linked to speech, language and communication difficulties ( Hammer *et al.*, 2017). Either way, once the child has been identified as having language and communication difficulties, early intervention is a critical and often involves training parents on how to promote early language development effectively in everyday interactions ( Barton & Fetting, 2013). This is because the long term negative educational, social and emotional consequences of having early speech and language difficulties early in development have been found to be improved through early intervention that focuses on parent-child interaction ( Armstrong *et al.*, 2017; Hammer *et al.*, 2017).

### Description of the intervention: Parent-child interaction therapy (PCIT)

Children develop within the context of their family and so parents and caregivers are best placed to support this development. Parent-child interaction therapy refers to a group of interventions mediated by trained parents to address areas of developmental difficulties in children. Although the intervention was originally developed to target challenging behaviour in children, it has since evolved to treat a range of difficulties, including speech language and communication ( Lieneman *et al.*, 2017). Language is acquired in everyday interactions between children and their parents, and as parents spend the most time interacting and communicating with their children, parent-child interaction therapy is considered to be ecologically valid and family-centred ( Barton & Fetting, 2013). The intervention is mediated through parents and caregivers by training them about the importance of responsivity and the quality and quantity of their language input in daily interactions and coaching them on strategies to implement this. There is now evidence that interventions mediated through parents may be as effective as those mediated through clinicians when delivered with sufficient quality ( Burgoyne *et al.*, 2018; Law *et al.*, 2003), are associated with improved outcomes in child language development ( Roberts *et al.*, 2019) and are recognised internationally as a valuable approach to remediating communication difficulties in young children ( Law *et al.*, 2019). We will use the term ‘parent’ in this paper to mean all caregivers who interact with children on a daily basis. Caregivers can include grandparents or other family caregiver who take on the ‘parent’ role for the purposes of the intervention. We will use the term parent-child interaction therapy to refer to those interventions targeting speech, language and communication difficulties in young children.

Parent-child interaction therapy (PCIT) is known by various names, including: ‘(interactive) focused stimulation’; ‘social-interaction therapy’; ‘responsive education/ teaching’; ‘naturalistic teaching’ or ‘milieu teaching’ ( O’Toole *et al.*, 2018). The aim of all programmes is to train parents to recognise and respond to verbal and nonverbal communication and interaction in their children in order to encourage an increase in these behaviours ( Warren *et al.*, 2008). One example of this intervention is the Hanen programme for parents ‘It Takes Two to Talk®’ ( Girolametto & Weitzman, 2006), which educates parents about the importance of child-oriented behaviours to promote joint attention and reciprocal interaction, and helps them to apply language facilitation strategies in natural, everyday interactions. This programme is delivered through classroom-based training of parents in groups in addition to individual coaching of parents with their children, but does not involve direct clinician-child intervention. Enhanced Milieu Teaching (EMT) or prelinguistic milieu teaching is another version of the intervention, which combines elements of responsivity education with behavioural strategies and milieu teaching through modelling and environmental arrangements in order to promote verbal and/or nonverbal language and communication ( Hancock & Kaiser, 2007). This intervention is mostly delivered intensively through one-to-one sessions and involves the clinician working directly with the child in addition to coaching the parent. Responsive Teaching is a relationship-based intervention that helps parents to engage in reciprocal interaction and respond contingently to their children’s behaviour with high levels of positive affect matched to their children’s development, interests, and behavioural style ( Mahoney & Perales, 2019). It focuses on the development of childhood pivotal behaviours in the areas of cognition (e.g. exploration and problem-solving), communication (e.g. vocalisation and joint attention) and social emotional functioning (e.g. empathy and cooperation). It can be delivered in groups, but most of the efficacy research has involved individual weekly one-hour sessions over a 3–6-month period. Similar to EMT, parents are first provided with a model of the desired strategy before being coached on their implementation by the clinician as they interact with their child. Finally, many programmes use an intervention known as ‘dialogic reading’ whereby parents are trained on how to read ‘with’ and not ‘to’ their child by engaging in active discussion and strategic questions when sharing a book ( Vally *et al.*, 2015).

### How the intervention might work

PCIT comes from social-interactionist/constructivist theories of language development, which state that children naturally learn to communicate based on how adults in their environment respond to and interact with them in daily activities ( Klatte *et al.*, 2019; Warren *et al.*, 2008). This theory holds that helping children to intentionally use broader and more frequent verbal and/or nonverbal means of communicating and by helping parents to recognise and respond to this in appropriate ways enhances language learning. The effect of this intervention is therefore bi-directional, in that children and adults reciprocally change how they interact as the child’s ability to communicate increases. The intervention is also in line with the transactional theory of development ( Sameroff & Fiese, 2000) which acknowledges the mutual effect of the child, caregiver and environment on a child’s developmental outcome. In the case of children with speech and language difficulties, it is assumed that communication may be more subtle than for typically developing children (e.g. through gestures, movements or vocalisations ( Pennington *et al.*, 2018) which can in turn affect how parents recognise and respond to their children. Therefore, this intervention works by training parents to be more aware of all means of communication and how to respond using naturalistic strategies with a greater frequency and intensity to help develop their children’s communication. When parents become more responsive to their children’s attempt to communicate and respond contingently to the child’s message, they improve the language learning environment. This is because any competing attentional and cognitive processing are reduced, allowing the child to focus on the appropriately delivered language input by the parent (for more see Levickis *et al.*, 2018). Pennington *et al.* (2018) and Oono *et al.* (2013) also note that additional benefits of the intervention may be to increase parents’ confidence and skills in how they communicate with their children as well as reduce parental stress and child frustration as communication becomes more successful for all. The aims of PCIT or parent-mediated interventions are as follows:

1. To foster and increase adult-child interaction and joint attention through child-centred activities.

2. To promote the frequency and complexity of adult responsivity to non-verbal and verbal communication.

3. To facilitate appropriate language modelling and prompting from adults that help the child to understand and produce language O’Toole *et al.*, 2018.

Roberts & Kaiser (2011) describe PCIT as ‘triadic’ as it involves the engagement of a clinician, parent and child. They also describe it as having a ‘cascading effect’ as an experienced clinician trains parents to use the interaction- and language-promoting strategies to a high degree of fidelity and consistency with their child, leading to enhanced language development in the child. This means that there are many aspects that can influence the overall effectiveness of the intervention, including the clinician’s experience, how the intervention is delivered, parental implementation of the strategies, and the child’s baseline language and cognitive skills and overall ( Roberts & Kaiser, 2011; Siller *et al.*, 2013). However, as the agent of change for the intervention is the parent, their engagement, reflection, understanding and acceptance of the intervention have a significant influence on the success of the programme.

### Parental experiences and perceptions of PCIT

Two recent papers have investigated the observations of Speech and Language Therapists (SLTs) about parental experiences with this intervention. Klatte *et al.* (2019) interviewed ten SLTs about their views on the facilitators and barriers towards parental engagement in PCIT for children with Developmental Language Disorder (DLD). They identified that the SLTs expected that they would reach a mutual understanding with parents about each other’s roles and expectations. The therapists also expected that they would create a constructive relationship though a supportive environment so that parents would feel empowered to understand their influence on the child’s progress. They also identified barriers to the intervention. These included physical (e.g. time, travel, childcare) and biopsychosocial (e.g. depression, illness and learning potential) issues. Davies *et al.* (2019) interviewed SLTs about their views on parental roles in intervention more broadly. Their findings indicated that SLTs see parents as a ‘helper’ and expect them to carry out home activities planned by the SLT. This is in contrast Klatte *et al.* (2019) paper that discussed parental ‘empowerment’. Although it could be argued that this was because Davies *et al.*, 2019 did not focus on PCIT, the views do go against the motivation behind parent-led intervention where the parent is a learner or adaptor of the intervention according to their own situation and their child’s changing development, and the SLT is in a coaching role.

The views of parents about their role in speech and language therapy intervention have also been reviewed in many studies. For example, Glogowska & Campbell (2000) found that parents do expect to have some role in intervention, but that this needs to made clear to them early on. This can be achieved through discussion about their perceptions, needs and concerns and then negotiated into what can be achieved with the therapist so as to avoid any misunderstanding. Parents often have different expectations about the therapy process, particularly that it will involve direct contact between the therapist and the child, and may not expect to be so heavily involved in the intervention themselves. For example, Baxendale *et al.* (2001) completed questionnaires followed by in-depth interviews involving eight parents who participated in traditional clinic-based therapy, and ten who took part in the Hanen Parent Programme focusing on PCIT. All of the children involved were aged between 30 and 42 months and had a diagnosis of language impairment. Parents in the PCIT group initially had difficulty accepting the philosophy behind this indirect approach, but later appreciated that it was more appropriate for children of this age. They also were able to attribute their child’s progress to changes they had made in their own interaction styles more than those in the clinic-based group. Although there were aspects of the intervention such as role play that were not viewed positively, the use of video feedback and support from other parents was welcomed. Davies *et al.* (2017) also interviewed parents about their role in SLT interventions and although parents were initially uncertain, as they gained greater experience they understood the importance of their role as an intervener. Similarly, Carroll (2010) reviewed parents’ expectations of speech and language therapy for their children with intellectual disability. It was noteworthy that parents in this study saw the therapist as the expert on their child in line with more of a medical model of treatment and expected the therapist to make decisions and carry out interventions to ‘fix’ their child’s speech difficulty. They were more ambivalent about their own role, indicating a possibly mismatch between their own expectations and that of the therapist.

Lyons *et al.* (2010) completed focus groups with parents about their expectations and experiences before and after engaging in an early intervention programme. Similar to previous studies, they noted that parents had expected to be guided about how to facilitate their child’s speech and language development but that their role would be more ‘observational’. They were therefore uncertain about why the activities were focused on their own behaviour instead of the child’s. Lyons *et al.* (2010) concluded that therapists need to move away from an ‘expert’ role, and engage in discussion with parents about their expectations before therapy starts so that they can work out what is to be involved together. This will ultimately affect parental perceptions and engagement with therapy. Finally, a recent qualitative systematised review looked at parental engagement in early speech-language pathology interventions (the term for speech and language therapy used in the US and Australia) ( Melvin *et al.*, 2019). ‘Engagement’ in this paper related to parental investment and involvement in therapy, which was found to be a complex process. The review found that parents need time and ongoing support in order to become empowered and engaged in early interventions. Furthermore, each parent engaged with the interventions in a unique way, and so time, open communication and trust building were identified as important aspects.

### Why is it important to do this synthesis?

Parental perceptions of this intervention are central to understanding the complex factors that make this intervention work. As discussed above, research has noted that parents do not always expect to have to be so heavily involved in their child’s treatment, but rather may assume that the SLT will ‘fix’ their child ( Carroll, 2010; Goodhue *et al.*, 2010). In addition, as the SLT will often not treat the child directly, the intervention is considered ‘indirect’, which can be confusing and unsatisfactory for parents ( Klatte & Roulstone, 2016). Although many quantitative systematic reviews exist on the effectiveness of this intervention ( Oono *et al.*, 2013; O’Toole *et al.*, 2018; Pennington *et al.*, 2018; Roberts & Kaiser, 2011; Roberts *et al.*, 2019; Zwi *et al.*, 2011), few have considered the qualitative evidence around the understanding, acceptability and implementation challenges that exist for parents. A recently published review by Melvin *et al.* (2019) did examine parental engagement in early interventions, but focused more broadly at the area of early intervention and not PCIT specifically. As PCIT requires parents to take on the role of the clinician and be coached in delivery of the intervention, parents are involved more heavily than in other interventions. Therefore, understanding the experiences of parents in this intervention in particular is worth investigating.

As outlined in the previous section, a number of qualitative studies have been published in this field. We need to gather and synthesize this evidence to describe parental experiences of this intervention using a systematic and rigorous approach. Integrating these findings with the previously published systematic reviews will provide a substantial evidence base for this intervention ( Schlosser, 2004). This will ultimately ensure that parents can be informed decision makers where they are provided with all of the relevant information so that they can actively collaboration with professionals and advocate for their children ( Crais *et al.*, 2006). The findings from the synthesis will be useful in guiding practice and policy for the implementation of PCIT, by capturing the parent voice in how they view their role in improving their child’s language and communication outcomes.

### Objectives

The aim of this review is to examine the experiences and perceptions of PCIT for parents of children with speech, language and communication difficulties using qualitative evidence synthesis. The objectives of the review are to:

1. Describe the experiences and perceptions of PCIT for parents of children with speech, language and communication difficulties. The intervention targets the language and interaction patterns of parents and their children in everyday interactions and can involve group and/or individual training. It can take place in community, clinical, preschool/ school or home-based settings. Interventions that involve telehealth or connected health technologies into the family home will also be included.
2. Examine the potential implications of the themes from the parental views that emerge from this synthesis for policy, regulation and practice in providing PCIT for children with communication difficulties.

## Protocol

This protocol has been submitted to the International Prospective Register of Systematic Reviews (PROSPERO) and this article will be updated with the identification number once the protocol has been accepted by PROSPERO.

### Criteria for considering studies for this synthesis

***Types of studies.*** We will consider primary research studies that use qualitative design methods such as ethnography, phenomenology, case studies and grounded theory for inclusion. The study design and analysis method (e.g. thematic analysis) must be clearly reported and must be qualitative to be included in the review. Mixed-methods studies will be included if it is possible to extract the qualitative data. All studies must be peer-reviewed articles. Studies which collect data qualitatively but analysed it quantitatively, and studies where the full text is not available will be excluded.

***Types of participants.*** Study participants will be parents and caregivers of children with any type of speech, language or communication difficulty. The children will have a range of conditions such as autism spectrum disorder, Down syndrome, Cerebral Palsy, Intellectual Disability, DLD or can be late-talking children with a communication difficulty of unknown origin. Studies will be included once the author(s) have stated that the children have a speech, language and communication difficulty or delay/disorder as diagnosed by a speech and language therapist. Children in the studies must be between the ages of birth to six year of age. There is the possibility of subgroup analysis within this population, for example analysis of perspectives of parents of children with autism spectrum disorder compared to children with other developmental/ communication difficulties; group vs. individually-delivered interventions or clinic vs. home-based interventions (see Subgroup Analysis below).

***Types of interventions.*** All types of PCIT interventions designed to improve the communication, interaction and language input of parents for their children with communication and language difficulties. The intervention will involve coaching, supervision and support from a clinician and will take place either on an individual or group basis. The interventions will take place in naturalistic contexts and focus on everyday interactions between parents and their children. If the intervention involves parent-mediated intervention delivered in conjunction clinician-mediated intervention, we will include it as long as the perspectives of parents is presented. However, we will exclude interventions where the clinician is the main provider of the intervention and parents are encouraged to do ‘home practice’ or ‘homework’ only, but no coaching is provided. We will also exclude interventions that focus only on speech sound production or stuttering/stammering as they tend to be more behavioural in their orientation. Finally, interventions that are parent-mediated but focus on other developmental aspects such as disruptive behaviour, motor development, social-emotional, cognitive or self-help skills will be excluded.

***Phenomenon of interest.*** The phenomenon of interest in this study is the experiences and perceptions of parents who take part in PCIT, which include studies of acceptability, engagement, understanding and importance of the intervention, facilitators and barriers to the intervention, parental role in changing their child’s language and communication, and any quality of life or stress indicators. Studies that focus on broader aspects of parenting children with communication difficulties will be excluded.

### Search methods for identification of studies

The review will systematically search the literature using electronic databases and also purposively sample papers using citation searching, contacting key authors and following up of references lists as outlined in Booth, (2016).

***Electronic searches***

We will search the following electronic databases:

- Scopus
- Web of Science
- EBSCO- CINAHL
- ERIC
- PsychINFO
- Embase
- Cochrane
- PubMed
- Academic Search Complete
- ProQuest Dissertations and Theses
- SpeechBITE

Using guidelines developed by the Cochrane Qualitative Research Methods Group for searching qualitative evidence ( Harris *et al.*, 2018), we will develop search strategies for each database in consultation with an expert librarian (DOD). We will not apply any limits on language, date or location. The search will be conducted by one author (COT) on all databases over one week.

A summary of the electronic search string is presented in Table 1. Certain terms will be truncated, for example parent* or child* to ensure all spellings are captured. We will adapt our searching of title and abstracts to the individual databases. We will report the results of searching, screening and included studies using the PRISMA flowchart ( Moher *et al.*, 2009).

**Table 1.** Search strings.

	experienc* OR perception*
AND	“parent-child interact*” OR focus*near/2 stimulation OR natural* near/2 teaching OR milieu near/2 teaching OR responsiv* near/2 education OR responsiv* near/2 teaching OR Hanen OR parent near/2 mediat* OR dialogic near/2 reading
AND	coach* OR educat* OR intervention* OR learn* OR program* OR teach* OR train* OR therap*
AND	parent* OR maternal* OR mother* OR father* OR paternal* OR carer* OR caregiver* OR care-giver*
AND	child* OR speech* or languag* or communicat*
AND	delay* OR disorder* OR disability* OR difficult* OR impair* OR need OR problem*
AND	qualitative OR “mixed*method*” OR narrative OR phenomenol* OR ethno* OR questionnaire OR “grounded theory” OR “case*study*” OR “action research” OR “focus group” OR thematic OR construction* OR hermeneutic OR heurist*

***Searching other resources.*** We will review the reference lists of all the included studies and key references. We will conduct a cited reference search of all included studies identified by the initial search using Google Scholar’s *Cited by* option ( Booth, 2016). We will contact authors of included studies to clarify reported published information and contact researchers with expertise relevant to the synthesis topic to request studies that might be eligible.

### Data collection, management and synthesis

***Selection of studies.*** We will import all references into Endnote X9 and remove duplicates. Two authors (COT and RL) will screen titles and abstracts independently to evaluate eligibility against our inclusion/exclusion criteria using Covidence systematic review management system. Where it is not possible to determine whether to include an article or not, the full text of the article will be retrieved. One author (COT) will review all full-text articles and another author (RL) will share second screening of all full-text articles. Disagreements between authors will be solved via discussion, or if required, in consultation with a third team member (CH). As necessary and appropriate, we will contact authors of potential included studies for further information. We will include a table listing the studies excluded from our synthesis at the full text stage and the main reasons for exclusion. We will collate multiple reports of the same study. We will report the outcome of the search strategy in a PRISMA flowchart ( Moher *et al.*, 2009).

***Sampling of studies.*** As qualitative evidence synthesis aims for variation in concepts rather than an exhaustive sample, and because large numbers of studies can affect the quality of the analysis, if the review retrieves more than 40 eligible papers, we will develop a sampling framework to ensure richness and representativeness, as used by Ames *et al.* (2019). This will involve extracting data from all studies found such as participant characteristics (e.g. mother/father, socioeconomic status and diagnosis of the child); study setting (e.g. home, clinic or tele-practice delivery); geographical location; type of PCIT (e.g. EMT or Hanen Parent Programme); data richness (quantity and quality of data provided) and study objectives in order to develop a sampling framework. Data richness will be assessed on a scale developed by EPOC, (2017). We will aim to ensure that the sample consists of a wide range of participants, rich data and a focus that resembles our synthesis objectives. We will report how we developed any sampling framework in the full review.

***Data extraction and management.*** We will import all full text articles into NVivo Version 12. Data extraction and the thematic synthesis will be facilitated within NVivo ( Houghton *et al.*, 2017). We will use categories such as author, year, location, study setting, sample characteristics (parents and children), intervention type, intervention setting, design, ethics, data collection and analysis methods, results/themes/findings including supporting quotations and sources of support.

***Data synthesis.*** Based on our consideration of the RETREAT criteria for selected qualitative evidence synthesis approaches, the review will synthesise the included qualitative studies using thematic synthesis ( Booth *et al.*, 2018). According to Thomas & Harden (2008), thematic synthesis is an inductive approach using three steps, which start with line by line coding of primary data from the included studies to develop descriptive themes and generate broader analytical themes. The primary data will include direct quotes from participants and the author interpretations of the data. This will be conducted within NVivo with guidance from a previous synthesis on how best to use the coding software ( Houghton *et al.*, 2017) and allows for transparency and clarity in the synthesis process. We chose thematic synthesis because it fits with the aim of this review, which is to provide information for policy and practice by presenting the voice of those who experience the intervention ( Barnett-Page & Thomas, 2009). One author (COT) will carry out the thematic synthesis with continuous input from two other authors (RL and CH) authors at each stage. All of the authors will read and make contributions to the final paper.

***Subgroup analysis.*** The potential for subgroup analysis will be determined inductively through the synthesis. Where there are sufficient numbers of studies found, this may include subgroup analysis based on the diagnosis of the children (e.g. children with autism vs. developmental language disorder); the nature of the intervention (group vs. individual) the setting in which it is delivered (clinic vs. home-based) or geographical location. It is generally middle-class, Western parents that participate in these studies, although more recently interventions have targeted at risk groups of families from lower socio-economic settings. Therefore, further subgroup analysis might look at the differences between those from lower and middle/upper socioeconomic groups as measured by maternal education and/or income levels as described by the studies.

***Assessment of methodological limitations in included studies.*** Two review authors (COT and RL) will independently assess the quality of the included studies using the Critical Appraisal Skills Programme (CASP) tool ( CASP, 2018). This tool examines a number of aspects of quality including the methodology, research design, data collection, relationship between researcher and participant, data analysis, findings and the value of the research among others. Although we will not exclude studies on the basis of their methodological limitations, this assessment will inform our overall confidence in the review findings.

***Assessment of confidence in the synthesis findings.*** Two authors (COT and CH) will use the GRADE CERQual (Confidence in the Evidence from Reviews of Qualitative research) approach to summarise our confidence in each finding ( Lewin *et al.*, 2018). This involves examining four main elements:

1. Methodological limitations of included studies.
2. Coherence of the review findings to the review question.
3. Adequacy of the data is in supporting the review finding.
4. Relevance of the included studies to the review question.

After assessing each of the four components, we will make a judgement about the overall confidence in the evidence supporting the review finding. We will judge confidence as high moderate, lower or very low. The final assessment will be based on consensus among the review authors. We will then include a sensitivity analysis to examine the contribution of the poorer quality studies to the overall findings ( Houghton *et al.*, 2017; Thomas & Harden, 2008).

### Reporting

This review will be reported in line with the ENTREQ guidelines ( Tong *et al.*, 2012). A completed PRISMA-P checklist is available from the Open Science Framework at https://www.doi.org/10.17605/OSF.IO/FDP3W ( O’Toole, 2019).

### Dissemination of findings

The findings will be submitted to a peer-reviewed journal for publication. They will also be integrated into a wider study that is currently planned to look at the feasibility of an intensive parent-child interaction therapy for Irish children with Down syndrome. The findings will also be shared with stakeholders, including parent and therapist groups via newsletters, social media and professional bodies.

### Study status

The study has not yet started.

## Discussion

Understanding the perspectives of parents in parent-child interaction therapy (PCIT) is important in deciphering facilitators and barriers to the behaviour change techniques that needs to take place in order to improve children’s speech, language and communication ( Justice *et al.*, 2015). The purpose of this protocol is to systematically assesses peer-reviewed qualitative studies of the perceptions of parents in implementing PCIT.

Furthermore, PCIT requires parents to alter how they talk to and interact with their children. It is therefore important to consider parental experiences and perceptions of the intervention in light of theories regarding the factors that might influence their implementation and maintenance. These include *Normalisation Process Theory* ( May *et al.*, 2009) which addresses how practices are put into action and embedded into everyday social routines and contexts and maintained over time. Furthermore, the *Theoretical Framework of Acceptability* ( Sekhon *et al.*, 2017) considers factors such as attitude, burden, perceived effectiveness, and self-efficacy which influence how people experience and respond to an intervention. Finally, a recent paper identified factors related the context, mechanism and outcomes that should be reviewed when looking at theories of collaborative work practices between parents and SLTs ( Klatte *et al.* (2020). It will be important to evaluate these theories in light of the evidence found in this synthesis.

The outcomes of this synthesis will add to the existing quantitative evidence for this intervention and give greater recognition to the parent’s perspectives which will untimely help to facilitate their involvement and increase their satisfaction with their child’s therapy ( Glogowska & Campbell, 2000). In this way the results should be included for future guidance for practice, regulation and policy in providing PCIT for all children with communication difficulties.

## Data availability

### Underlying data

No data is associated with this article.

### Reporting guidelines

Open Science Framework: PRISMA-P checklist for ‘The experiences and perceptions of parent-child interaction therapy for parents of young children with communication difficulties: A qualitative evidence synthesis protocol’, https://www.doi.org/10.17605/OSF.IO/FDP3W ( O’Toole, 2019).

Data are available under the terms of the Creative Commons Zero “No rights reserved” data waiver (CC0 1.0 Public domain dedication).
