# Supplementary material for: Stage 1 Registered Report: The experiences and perceptions of parent-child interaction therapy for parents of young children with communication difficulties: A qualitative evidence synthesis protocol
Source: HRB Open Res. 2020 May 20;2:36. Originally published 2019 Dec 16. [Version 2] doi: 10.12688/hrbopenres.12974.2 (PMC7281664; doi:10.12688/hrbopenres.12974.2)
Supplement: Peer review history

# Peer review: Reviewer response for version 2

**DOI:** 10.21956/hrbopenres.14161.r27430

**Published:** 2020-06-08

**Authors:** Lindsay Pennington

**Affiliations:** 1 Institute of Health and Society, Newcastle University, Newcastle upon Tyne, UK

The revised article is very clearly presented and well argued. I look forward to reading the results of the study in due course.

Is the study design appropriate for the research question?

Yes

Have the authors pre-specified sufficient outcome-neutral tests for ensuring that the results obtained can test the stated hypotheses, including positive controls and quality checks?

Yes

Is the rationale for, and objectives of, the study clearly described?

Yes

Are sufficient details of the methods provided to allow replication by others?

Partly

Are the datasets clearly presented in a useable and accessible format?

Yes

Reviewer Expertise:

NA

I confirm that I have read this submission and believe that I have an appropriate level of expertise to confirm that it is of an acceptable scientific standard.

# Peer review: Reviewer response for version 2

**DOI:** 10.21956/hrbopenres.14161.r27429

**Published:** 2020-05-29

**Authors:** Rae Thomas

**Affiliations:** 1 Institute for Evidence-Based Healthcare, Bond University, Gold Coast, Australia

The authors have done a good job at addressing my queries and articulating the definition of PCIT in the field of speech and language therapy. 

 Although I agree that views of intervention participants (in this case parents) should inform policy and practice I am less convinced they often do. Does the reference for Barnett-Page and Thomas, 2009 report policies that have been modified because of participant views or is it theoretical? If the former, it would be good, in the discussion to demonstrate how this might be achieved in concrete rather than abstract forms, particularly if this is an objective. 

 Thank you for the opportunity again to review the manuscript. I believe it is fine for indexing.

Is the study design appropriate for the research question?

Yes

Have the authors pre-specified sufficient outcome-neutral tests for ensuring that the results obtained can test the stated hypotheses, including positive controls and quality checks?

Yes

Is the rationale for, and objectives of, the study clearly described?

Partly

Are sufficient details of the methods provided to allow replication by others?

Yes

Are the datasets clearly presented in a useable and accessible format?

Not applicable

Reviewer Expertise:

Psychology, implementing behaviour management interventions, conducting systematic and scoping reviews.

I confirm that I have read this submission and believe that I have an appropriate level of expertise to confirm that it is of an acceptable scientific standard.

# Response

**Published:** 2020-06-04

**Authors:** Ciara O'Toole

**Affiliations:** University College Cork, Ireland

Thank you for your comments on our revised paper and for approving the same. In relation to the query about  Barton-Page and Thomas (2009) review of methods for synthesising qualitative research, this was commissioned by the Department of Health in the UK to inform policy in practice. In this case, it is more theoretical- presenting the evidence of using thematic synthesis in a way "that could inform" policy although as you point out, there are as yet no concrete examples of this.

# Peer review: Reviewer response for version 1

**DOI:** 10.21956/hrbopenres.14059.r27340

**Published:** 2020-05-07

**Authors:** Rae Thomas

**Affiliations:** 1 Institute for Evidence-Based Healthcare, Bond University, Gold Coast, Australia

**Overall comment**: Thank you for the opportunity to review this manuscript. There is thoroughness in the proposed review method, however the definition of the intervention is confusing.

 The authors appear to take a broad definition of Parent-Child Interaction Therapy (PCIT) and appear to encompass a range of different therapies. However, I also note the authors use the name PCIT as an actual intervention (“PCIT is **an** early intervention”; As the success of **the** intervention” etc). PCIT is a behavioural intervention developed in the 1980s for children with behavioural challenges, not specifically for children with communication difficulties and their parents ( http://www.pcit.org). It would conceivably work for children with these challenges and their parents but it was not developed for that purpose. Confusion regarding the specific intervention known as PCIT versus interventions that proritise and intervene in parent-child interactions is underscored by not including any references from the intervention known as PCIT. This needs to be clarified and is my major concern with the protocol and is reiterated in several sections of my review. I note the authors have published a Cochrane SR on “Parent-mediated interventions for promoting communication and language development in young children with Down syndrome” and some references are the same. Is this what is meant by PCIT?

 Given the broad focus of the research study (the long list of phenomenon of interest, page 6), it might be better to consider this a scoping review.

**Title: **

1. It is convention for the study design to be part of the title. Do the authors intend for this to be a systematic review? It is set up like one and has it as a key word but it is not mentioned in either the title or abstract.

**Abstract**:

1. The authors will need to clarify what they mean by PCIT throughout the manuscript. This is the major concern I have with the manuscript in its present format.
2. The first sentence of the Methods part of the abstract is not necessary there because it is an aim and not a method.
3. In the Discussion section of the abstract, what do the authors mean be “help guide best practice and policy”? How do the authors think experiences and perceptions of people involved in PCIT will guide these two areas?

**Introduction**:

1. Please define PCIT. It is very different to the therapy conventionally known as PCIT.
2. References are missing in some areas For example, “parent-child interaction therapy is considered to be ecologically valid and family-centred” (page 3) and “Parent-child interaction therapy (PCIT) is known by various names, including xxx, xxx etc”. I note the authors have published a Cochrane SR on parent-mediated interventions for promoting communication and language development. Is this what they mean by PCIT? If so, please use this term consistently.
3. There is no documentation as to how the authors intend to meet Objective 2 “Examine the potential implications of this synthesis for policy, regulation and practice in providing PCIT for children with communication difficulties”. Please describe how this is to be done in the Data collection, management and synthesis section.

**Protocol**:

1. The authors state: “Finally, interventions that are parent-mediated but focus on other developmental aspects such as disruptive behaviour, motor development or self-help skills will be excluded.” What do the authors mean when they say “other” developmental aspects? What developmental focus do they intend to include?
2. Normally I would suggest the large number of databases is unnecessary but as the authors have already conducted a Cochrane review with these databases, I assume they have a fair idea of the number of articles they need to screen. Having said that this review is more broad than Down syndrome.
3. In the data synthesis section, what will you extract? The quotes from the papers or the interpretation of those quotes to a framework? Should I assume “primary data” are the actual quotes?
4. I think the heading “Subgroup analysis and heterogeneity” should be modified as this typically relates to quantitative analyses. I would caution the authors in doing too many “subgroups” as the included number of articles is likely small.

**Discussion**:

1. Please describe how this will provide guidance for regulation and policy.

Thank you.

Is the study design appropriate for the research question?

Yes

Have the authors pre-specified sufficient outcome-neutral tests for ensuring that the results obtained can test the stated hypotheses, including positive controls and quality checks?

Yes

Is the rationale for, and objectives of, the study clearly described?

Partly

Are sufficient details of the methods provided to allow replication by others?

Yes

Are the datasets clearly presented in a useable and accessible format?

Not applicable

Reviewer Expertise:

Psychology, implementing behaviour management interventions, conducting systematic and scoping reviews.

I confirm that I have read this submission and believe that I have an appropriate level of expertise to confirm that it is of an acceptable scientific standard, however I have significant reservations, as outlined above.

# Response

**Published:** 2020-05-14

**Authors:** Ciara O'Toole

**Affiliations:** University College Cork, Ireland

**General Response:**

We would like to thank our reviewers for their detailed and thorough feedback. We have made a number of changes to the manuscript following this feedback, and hope that it has improved the clarity and quality of our protocol. The most substantive changes were adding more information on the theoretical underpinnings to the intervention, clarifying terminology and issues raised regarding the methodology.

We have responded to each reviewer’s comment in turn using the following codes:

*R1 = reviewer 1; R2= Reviewer 2; C1 = comment 1 etc.*

**R2CI:** The authors appear to take a broad definition of Parent-Child Interaction Therapy (PCIT) and appear to encompass a range of different therapies. However, I also note the authors use the name PCIT as an actual intervention (“PCIT is an early intervention”; As the success of the intervention” etc). PCIT is a behavioural intervention developed in the 1980s for children with behavioural challenges, not specifically for children with communication difficulties and their parents ( http://www.pcit.org). It would conceivably work for children with these challenges and their parents but it was not developed for that purpose. Confusion regarding the specific intervention known as PCIT versus interventions that proritise and intervene in parent-child interactions is underscored by not including any references from the intervention known as PCIT. This needs to be clarified and is my major concern with the protocol and is reiterated in several sections of my review. I note the authors have published a Cochrane SR on “Parent-mediated interventions for promoting communication and language development in young children with Down syndrome” and some references are the same. Is this what is meant by PCIT?

Given the broad focus of the research study (the long list of phenomenon of interest, page 6), it might be better to consider this a scoping review.

**Response: **In the field of speech and language therapy, the term PCIT is the overarching term used to refer to a number of interventions that are mediated by trained parents to improve the speech, language and communication of children. However we appreciate that the term is also used in other fields, particularly those used to target challenging behaviour. We have therefore acknowledged the various uses of the term in our abstract and introduction and clarified what we mean by PCIT in the context of this review. The focus of the review is therefore only on PCIT that targets language and communication and so we will continue to explore it as a qualitative evidence synthesis.

**R2 C2:** Title: It is convention for the study design to be part of the title. Do the authors intend for this to be a systematic review? It is set up like one and has it as a key word but it is not mentioned in either the title or abstract.

**Response: **This is a qualitative evidence synthesis, which is an umbrella term for the methodologies associated with the systematic review of qualitative research evidence (Carroll, 2017) and so it is important that this is retained in title. It is also in the methods section of the abstract. This is now clarified in the body of the text.

**R2 C3:** Abstract: The authors will need to clarify what they mean by PCIT throughout the manuscript. This is the major concern I have with the manuscript in its present format.

**Response: **This term is now explained in the abstract and introduction.

**R2 C4:** The first sentence of the Methods part of the abstract is not necessary there because it is an aim and not a method.

**Response:** As qualitative evidence synthesis is the method, it has been retained in this sentence, and the term ‘qualitative’ added.

**R2 C5:** In the Discussion section of the abstract, what do the authors mean by “help guide best practice and policy”? How do the authors think experiences and perceptions of people involved in PCIT will guide these two areas?

**Response: **Parental views should form part of best practice and policy. This sentence has been rewritten to clarify this. We have also expanded this in the section *Data synthesis* as thematic synthesis is regarded as a useful intervention for informing policy.

**R2 C6:** Introduction: Please define PCIT. It is very different to the therapy conventionally known as PCIT.

**Response: **We have now defined what we mean by PCIT in the context of speech and language therapy interventions, acknowledging that it can be used for other areas of behaviour.

**R2 C7:** References are missing in some areas For example, “parent-child interaction therapy is considered to be ecologically valid and family-centred” (page 3) and “Parent-child interaction therapy (PCIT) is known by various names, including xxx, xxx etc”. I note the authors have published a Cochrane SR on parent-mediated interventions for promoting communication and language development. Is this what they mean by PCIT? If so, please use this term consistently.

**Response:** References now added. We have clarified what is meant by PCIT in the introduction and is the same meaning that was taken in the Cochrane review. More recent descriptions of the intervention have started to use the term PCIT (e.g. Klatte et al., 2019 as referenced in this paper).

**R2 C8:** There is no documentation as to how the authors intend to meet Objective 2 “Examine the potential implications of this synthesis for policy, regulation and practice in providing PCIT for children with communication difficulties”. Please describe how this is to be done in the Data collection, management and synthesis section.

**Response: **Thematic synthesis is known to help inform policy and practice. We have highlighted this in the Data synthesis section with the sentence: *We chose thematic synthesis because it fits with the aim of this review, which is to provide information for policy and practice by presenting the voice of those who experience the intervention (* *Barnett-Page & Thomas, 2009* *).*

**R2 C9:** Protocol: The authors state: “Finally, interventions that are parent-mediated but focus on other developmental aspects such as disruptive behaviour, motor development or self-help skills will be excluded.” What do the authors mean when they say “other” developmental aspects? What developmental focus do they intend to include?

**Response: **The other areas of development are those listed and social-emotional and cognitive development which have been added. The only focus will be on speech, language and communication. Anything outside of this will be excluded.

**R2 C10:** Normally I would suggest the large number of databases is unnecessary but as the authors have already conducted a Cochrane review with these databases, I assume they have a fair idea of the number of articles they need to screen. Having said that this review is more broad than Down syndrome.

**Response: **We have retained the planned search strategy to ensure that a wide range of papers is captured.

**R2 C11:** In the data synthesis section, what will you extract? The quotes from the papers or the interpretation of those quotes to a framework? Should I assume “primary data” are the actual quotes?

**Response: **This has been clarified with the sentence: *The primary data will include direct quotes from participants and the author interpretations of the data*

**R2 C12:** I think the heading “Subgroup analysis and heterogeneity” should be modified as this typically relates to quantitative analyses. I would caution the authors in doing too many “subgroups” as the included number of articles is likely small.

**Response: **This heading has been changed to ‘ *subgroup analysis’*. A caveat of not including too many subgroups is provided.

**R2 C13:** Discussion: Please describe how this will provide guidance for regulation and policy.

**Response: **The link between including parental perspectives for future iterations of policy has been added in the final sentence.

# Peer review: Reviewer response for version 1

**DOI:** 10.21956/hrbopenres.14059.r27090

**Published:** 2020-02-11

**Authors:** Lindsay Pennington

**Affiliations:** 1 Institute of Health and Society, Newcastle University, Newcastle upon Tyne, UK

This study will examine parents’ perceptions and experiences of training to facilitate their child’s language and communication development provided by speech and language therapists. This type of intervention is often used in clinical practice, but varies in the methods of delivery, content and dosage. The effectiveness of the interventions is yet to be fully determined. This review will provide timely and important information to guide clinical practice and further research.

 The protocol is clearly written and the design of the review to be conducted is well thought through. The search strategy is appropriate and comprehensive. The analysis plan is appropriate. The authors should state if analysis will be inductive or both inductive and deductive. Theories of factors influencing implementation of interventions could be helpful in assessing factors that may influence parents’ experiences and perceptions of the intervention, highlighting factors that have been raised in research to date and those that may be influential but have not yet been assessed.  (e.g.  May *et al. (*2009) ^1^;  Sekhon *et al. *(2017) ^2^).

 Please state the characteristics on which studies will be sampled if over 40 fit the review criteria.

 Subgroup analysis could include setting – at least one of the named interventions has been delivered outside North America, Europe and Australia (where the bulk of the research takes place). How will socio-economic group be defined?

 I have some minor suggestions for changes to the text:

- In the first section of the Background, what is the evidence for parent-child interaction training for children ‘at risk’ versus children with diagnosed speech, language or communication difficulty?
- N.B. children with biomedical conditions such as Down syndrome are not always diagnosed early.
- Description of the intervention: Explain the meaning of ‘pivotal behaviours’ and add a supporting reference.
- How the intervention may work: The authors could provide more detail on how increased parental responsivity may facilitate children’s communication. Theories of communication and language development could be added here.
- Type of Participants: cerebral palsy, Down syndrome etc. are not ‘types of speech, language or communication difficulty’. The first part of this section should be rewritten.
- ‘Goldbard’ should be Goldbart.
- Speech and language therapy is used in the main to describe the workforce, but on P5 ‘speech pathology’ is referred to. It would be helpful to explain this additional term.
- There are a few typos in the protocol and it requires some more proof reading.

Is the study design appropriate for the research question?

Yes

Have the authors pre-specified sufficient outcome-neutral tests for ensuring that the results obtained can test the stated hypotheses, including positive controls and quality checks?

Yes

Is the rationale for, and objectives of, the study clearly described?

Yes

Are sufficient details of the methods provided to allow replication by others?

Partly

Are the datasets clearly presented in a useable and accessible format?

Yes

Reviewer Expertise:

NA

I confirm that I have read this submission and believe that I have an appropriate level of expertise to confirm that it is of an acceptable scientific standard, however I have significant reservations, as outlined above.

## References

[1] : Development of a theory of implementation and integration: Normalization Process Theory. *Implement Sci*.2009;4: 10.1186/1748-5908-4-29 29 10.1186/1748-5908-4-29 19460163PMC2693517

[2] : Acceptability of healthcare interventions: an overview of reviews and development of a theoretical framework. *BMC Health Services Research*.2017;17(1) : 10.1186/s12913-017-2031-8 10.1186/s12913-017-2031-8 28126032PMC5267473

# Response

**Published:** 2020-05-14

**Authors:** Ciara O'Toole

**Affiliations:** University College Cork, Ireland

**General Response:**

We would like to thank our reviewers for their detailed and thorough feedback. We have made a number of changes to the manuscript following this feedback, and hope that it has improved the clarity and quality of our protocol. The most substantive changes were adding more information on the theoretical underpinnings to the intervention, clarifying terminology and issues raised regarding the methodology.

We have responded to each reviewer’s comment in turn using the following codes:

*R1 = reviewer 1; R2= Reviewer 2; C1 = comment 1 etc.*

**R1C1:** The authors should state if analysis will be inductive or both inductive and deductive.

**Response:** We have clarified that the analysis is inductive, and amended the working on page 7 to be “Thematic synthesis is an *inductive* approach using three steps”.

**R1C2:** Theories of factors influencing implementation of interventions could be helpful in assessing factors that may influence parents’ experiences and perceptions of the intervention, highlighting factors that have been raised in research to date and those that may be influential but have not yet been assessed.  (e.g.  May et al. (2009) 1;  Sekhon et al. (2017) 2).

**Response:** Thank you for directing us to this literature. It will be very relevant when reviewing our findings from the synthesis and taking them forward. We have included reference to these studies and the theories on factors that influence implementation of these interventions on page 7 under ‘Discussion. However as we are not using a framework synthesis which involves a more deductive approach we will not be analysing our results to an existing theoretical model.

**R1C3:** Please state the characteristics on which studies will be sampled if over 40 fit the review criteria.

**Response**: We have added greater detail on this based on Ames criteria under ‘sampling of studies’ and referring to the to the data richness scale as developed by the Cochrane Effective Practice and Organisation of Care (EPOC) group. We have included the following text: *This will involve extracting data from all studies found such as participant characteristics (e.g. mother/father, socioeconomic status and diagnosis of the child); study setting (e.g. home, clinic or tele-practice delivery); geographical location; type of PCIT (e.g. EMT or Hanen Parent Programme); data richness (quantity and quality of data provided) and study objectives in order to develop a sampling framework. Data richness will be assessed on a scale developed by EPOC (2017). We will aim to ensure that the sample consists of a wide range of participants, rich data and a focus that resembles our synthesis objectives* *. We will report how we developed any sampling framework in the full review.*

**R1C4:** Subgroup analysis could include setting – at least one of the named interventions has been delivered outside North America, Europe and Australia (where the bulk of the research takes place). How will socio-economic group be defined?

**Response: **We have added geographical location as a potential subgroup analysis, and clarified that SES will be defined by maternal education and/or income level as described in the studies.

**R1C5:** In the first section of the Background, what is the evidence for parent-child interaction training for children ‘at risk’ versus children with diagnosed speech, language or communication difficulty?

**Response: **The terms ‘at risk’ has now been removed and clarification added that early intervention is important for all children with language and communication difficulties.

**R1C6:** N.B. children with biomedical conditions such as Down syndrome are not always diagnosed early.

**Response: **We have clarified to say that they are ‘often’ diagnosed early in development to acknowledge that this does not always happen.

**R1C7:** Description of the intervention: Explain the meaning of ‘pivotal behaviours’ and add a supporting reference.

**Response: **Examples of pivotal behaviours are now added in the sentence **“**It focuses on the development of childhood pivotal behaviours in the areas of cognitive (e.g. exploration and problem-solving), communication (e.g. vocalisation and joint attention) and social emotional functioning (e.g. empathy and cooperation).” Supporting reference of related manual for the intervention (Mahoney & Perales, 2019) is now provided.  

**R1C8:** How the intervention may work: The authors could provide more detail on how increased parental responsivity may facilitate children’s communication. Theories of communication and language development could be added here.

**Response: **Further information on how parental responsibility facilitates communication is provided under ‘How the intervention may work’. Theories such as social interactionist/constructive and views of language development and the transactional theory of development are explored.

**R1C9:** Type of Participants: cerebral palsy, Down syndrome etc. are not ‘types of speech, language or communication difficulty’. The first part of this section should be rewritten.

**Response: **This section has now been re-written

**R1C10:** ‘Goldbard’ should be Goldbart.

**Response:** Amended

**R1C11:** Speech and language therapy is used in the main to describe the workforce, but on P5 ‘speech pathology’ is referred to. It would be helpful to explain this additional term.

**Response:** This is the terms for speech and language therapy used in the US and Australia. This explanation has been added

**R1C12:** There are a few typos in the protocol and it requires some more proof reading.

**Response:** The protocol has been full proofread.
